# Supplementary material for: Resolving the full spectrum of human genome variation using Linked-Reads
Source: Genome Res. 2019 Apr;29(4):635–45. doi: 10.1101/gr.234443.118 (PMC6442396; doi:10.1101/gr.234443.118)
Supplement: Supplemental Material [file supp_gr.234443.118_Supplemental_longranger-2.2.zip › longranger-2.2.tar.bz2!/lr2.2-public/lib/stan/cmdstan/stan/doc/api/html/classstan_1_1math_1_1accumulator.html]

Stan: stan::math::accumulator< T > Class Template Reference


|  |  |
| --- | --- |
|  | Stan  2.5.0  probability, sampling & optimization |


- Main Page
- Related Pages
- Namespaces
- Classes
- Files

- Class List
- Class Index
- Class Hierarchy
- Class Members

All Classes Namespaces Files Functions Variables Typedefs Enumerations Enumerator Friends Macros Pages

- stan
- math
- accumulator

Public Member Functions |
List of all members

stan::math::accumulator< T > Class Template Reference

Class to accumulate values and eventually return their sum.
More...

`#include <accumulator.hpp>`

|  |  |
| --- | --- |
| Public Member Functions | |
|  | accumulator () |
|  | Construct an accumulator. More... |
|  | |
|  | ~accumulator () |
|  | Destroy an accumulator. More... |
|  | |
| template<typename S > | |
| boost::enable\_if  < boost::is\_arithmetic< S >  , void >::type | add (S x) |
|  | Add the specified arithmetic type value to the buffer after static casting it to the class type `T`. More... |
|  | |
| template<typename S > | |
| boost::disable\_if  < boost::is\_arithmetic< S >  , typename boost::enable\_if  < boost::is\_same< S, T >, void >  ::type >::type | add (const S &x) |
|  | Add the specified non-arithmetic value to the buffer. More... |
|  | |
| template<typename S , int R, int C> | |
| void | add (const Eigen::Matrix< S, R, C > &m) |
|  | Add each entry in the specified matrix, vector, or row vector of values to the buffer. More... |
|  | |
| template<typename S > | |
| void | add (const std::vector< S > &xs) |
|  | Recursively add each entry in the specified standard vector to the buffer. More... |
|  | |
| T | sum () const |
|  | Return the sum of the accumulated values. More... |
|  | |

## Detailed Description

### template<typename T> class stan::math::accumulator< T >

Class to accumulate values and eventually return their sum.

If no values are ever added, the return value is 0.

This class is useful for speeding up auto-diff of long sums because it uses the `sum()` operation (either from `stan::math` or one defined by argument-dependent lookup.

Template Parameters
:   |  |  |
    | --- | --- |
    | T | Type of scalar added |

Definition at line 25 of file accumulator.hpp.

## Constructor & Destructor Documentation

template<typename T>

|  |  |  |  |  |
| --- | --- | --- | --- | --- |
| stan::math::accumulator< T >::accumulator | ( |  | ) |  |

inline

Construct an accumulator.

Definition at line 33 of file accumulator.hpp.

template<typename T>

|  |  |  |  |  |
| --- | --- | --- | --- | --- |
| stan::math::accumulator< T >::~accumulator | ( |  | ) |  |

inline

Destroy an accumulator.

Definition at line 40 of file accumulator.hpp.

## Member Function Documentation

template<typename T>

template<typename S >

|  |  |  |  |  |  |
| --- | --- | --- | --- | --- | --- |
| boost::enable\_if<boost::is\_arithmetic<S>, void>::type stan::math::accumulator< T >::add | ( | S | *x* | ) |  |

inline

Add the specified arithmetic type value to the buffer after static casting it to the class type `T`.

See the Boost doc for `boost::is_arithmetic` for information on what counts as an arithmetic type.

Template Parameters
:   |  |  |
    | --- | --- |
    | S | Type of argument |

Parameters
:   |  |  |
    | --- | --- |
    | x | Value to add |

Definition at line 54 of file accumulator.hpp.

template<typename T>

template<typename S >

|  |  |  |  |  |  |
| --- | --- | --- | --- | --- | --- |
| boost::disable\_if<boost::is\_arithmetic<S>, typename boost::enable\_if<boost::is\_same<S,T>, void>::type >::type stan::math::accumulator< T >::add | ( | const S & | *x* | ) |  |

inline

Add the specified non-arithmetic value to the buffer.

This function is disabled if the type `S` is arithmetic or if it's not the same as `T`.

See the Boost doc for `boost::is_arithmetic` for information on what counts as an arithmetic type.

Template Parameters
:   |  |  |
    | --- | --- |
    | S | Type of argument |

Parameters
:   |  |  |
    | --- | --- |
    | x | Value to add |

Definition at line 74 of file accumulator.hpp.

template<typename T>

template<typename S , int R, int C>

|  |  |  |  |  |  |
| --- | --- | --- | --- | --- | --- |
| void stan::math::accumulator< T >::add | ( | const Eigen::Matrix< S, R, C > & | *m* | ) |  |

inline

Add each entry in the specified matrix, vector, or row vector of values to the buffer.

Template Parameters
:   |  |  |
    | --- | --- |
    | S | type of values in matrix |
    | R | number of rows in matrix |
    | C | number of columns in matrix |

Parameters
:   |  |  |
    | --- | --- |
    | m | Matrix of values to add |

Definition at line 88 of file accumulator.hpp.

template<typename T>

template<typename S >

|  |  |  |  |  |  |
| --- | --- | --- | --- | --- | --- |
| void stan::math::accumulator< T >::add | ( | const std::vector< S > & | *xs* | ) |  |

inline

Recursively add each entry in the specified standard vector to the buffer.

This will allow vectors of primitives, auto-diff variables to be added; if the vector entries are collections, their elements are recursively added.

Template Parameters
:   |  |  |
    | --- | --- |
    | S | Type of value to recursively add. |

Parameters
:   |  |  |
    | --- | --- |
    | xs | Vector of entries to add |

Definition at line 103 of file accumulator.hpp.

template<typename T>

|  |  |  |  |  |
| --- | --- | --- | --- | --- |
| T stan::math::accumulator< T >::sum | ( |  | ) | const |

inline

Return the sum of the accumulated values.

Returns
:   Sum of accumulated values.

Definition at line 113 of file accumulator.hpp.

---

The documentation for this class was generated from the following file:

- src/stan/math/matrix/accumulator.hpp


---

[ Stan Home Page ]

*© 2011–2014,
Stan Development Team.*
